# Short-Term and Medium-Term Radiological and Clinical Assessment of Patients with Symptomatic Flexible Flatfoot Following Subtalar Arthroereisis with Spherus Screw

**DOI:** 10.3390/jcm12155038

**Published:** 2023-07-31

**Authors:** Andrzej Bobiński, Łukasz Tomczyk, Paweł Reichert, Piotr Morasiewicz

**Affiliations:** 1 Department of Orthopaedic and Trauma Surgery, Institute of Medical Sciences, University of Opole, Witosa 26, 45-401 Opole, Poland; bobinski@interia.eu; 2 Department of Food Safety and Quality Management, Poznan University of Life Sciences, Wojska Polskiego 28, 60-637 Poznan, Poland; tomczyk@up.poznan.pl; 3 Department of Trauma and Hand Surgery, Wroclaw Medical University, Borowska 213, 50-556 Wroclaw, Poland; pawel.reichert@umed.wroc.pl

**Keywords:** flexible flatfoot, pes planovalgus, arthroereisis, symptomatic flatfoot, radiological, clinical

## Abstract

Background: There have been no reports on arthroereisis screw insertion into the talus in patients with flexible flatfoot. We aimed to conduct a clinical and radiological assessment in patients with symptomatic pes planovalgus deformity treated with a talar screw. Methods: This study involved a prospective assessment of 27 patients treated surgically for symptomatic flexible flatfoot deformity in the period 2021–2022. The following parameters were assessed in this study: Meary’s angle, the Costa–Bartani angle, the calcaneal pitch angle, surgery duration, the length of hospital stay, patient satisfaction, patients’ retrospective willingness to consent to the treatment they received, postoperative complications, and the use of analgesics. Results: The mean follow-up period was 14.76 months. Meary’s angle decreased from 18.63° before surgery to 9.39° at follow-up (*p* = 0.004). The Costa–Bartani angle decreased significantly from 154.66° before surgery to 144.58° after surgery (*p* = 0.012). The calcaneal pitch angle changed from 16.21° before to 19.74°. Complications were reported in three patients (11.11%). The mean surgery duration was 32 min. The mean hospital stay was 2.2 days. Fourteen patients (51.85%) were highly satisfied with the treatment, and 12 patients (44.44%) were quite satisfied with treatment. Twenty-five (92.59%) of the evaluated patients would choose the same type of treatment again. Six patients (22.22%) needed to use analgesics prior to surgical treatment, whereas none of the patients needed to use them by the final follow-up. Conclusion: Spherus screw arthroereisis helps improve radiological parameters in patients with flexible flatfoot. We observed good clinical outcomes after treatment with a talar screw, with a majority of patients reporting moderate-to-high levels of satisfaction with treatment. Both short- and medium-term treatment outcomes of pes planovalgus treatment with the use of Spherus screw are good.

## 1. Introduction

A flatfoot (pes planovalgus) is a common problem encountered by orthopedic surgeons [1,2,3,4,5,6,7,8,9,10,11,12,13,14,15,16,17,18,19,20,21,22]. Depending on the evaluated population, the estimated prevalence of pes planovalgus ranges from 2.7% to 59% [1,5,6,14,15]. Asymptomatic pes planovalgus does not require surgical treatment. However, symptomatic flatfoot may produce pain in the tarsal sinus and medial part of the foot, leg, and knee, limping or other gait disturbances, and limited exercise capacity, which may be associated with problems with footwear, daily activities, and sports [1,2,5,6,7,10,11,13,14,15,16,17,18]. Symptomatic pes planovalgus with accompanying pain, which affects approximately 2–14.2% of all flatfoot cases, is an indication for surgical treatment [1,2,3,4,5,6,7,10,11,12,14,15,16,17,18,19,20,21,22]. There are a number of approaches used in the treatment of symptomatic pes planovalgus [1,2,3,4,5,6,7,8,9,10,11,12,13,14,15,16,17,18,19,20,21,22]. One technique, subtalar arthroereisis (a minimally invasive propping up of the talocalcaneal joint), involves the use of expandable sinus tarsi implants or calcaneal screws. Another technique involves introducing a fibular bone graft into the space between the calcaneus and talus. There are also corrective procedures involving calcaneal or talar wedge osteotomy, tendon transfer, tendon lengthening, gastrocnemius muscle or Achilles’ tendon release, and subtalar, talonavicular, or calcaneocuboid arthrodesis [1,2,3,4,5,6,7,8,9,10,11,12,13,14,15,16,17,18,19,20,21,22]. Earlier arthoereisis techniques involved the insertion of various screws into the calcaneus or the placement of an expandable implant into the sinus tarsi [1,2,4,5,6,7,8,9,10,11,14,15,16,17,18,19,20,21,22]. Until recently, there have been no reports on arthroereisis screw insertion into the talus. Marketed several years ago, the Spherus talar screw (Gruppo Bioimpianti S.R.L., Italy) had been designed as a sinus tarsi plug to be introduced through the sinus tarsi into the talus. This is a novel surgical technique, whose outcomes have not yet been reported in the literature. Viewed from a biomechanical perspective, the Spherus screw is a self-locking wedge implant that, once placed in the talus, limits hindfoot eversion.

Apart from pain reduction, pes planovalgus treatment aims to improve foot function, radiological parameters, and gait by ensuring a physiological position of the foot under weight-bearing conditions [2,5,6,7,8,9,10,12].

Pes planovalgus treatment requires both functional and radiological assessments [1,2,3,4,5,7,8,9,10,14,15,16,17,18,19,22]. There has been no consensus on the preferred type of implant to be used for arthroereisis [1,2,3,4,5,6,7,8,9,10,11,12,13,14,15,16,17,18,19,20,21,22]. Earlier studies evaluated selected radiological and functional parameters following surgical treatment with the use of calcaneal implants [1,2,4,9,10,20,21], expandable sinus tarsi implants [5,8,14,15,16,19,20,21], or a fibular bone graft inserted between the talus and calcaneus [3]. Li et al., who evaluated 30 patients, reported good clinical and radiological outcomes following arthroereisis combined with soft-tissue procedures [17]. Vogt, who compared three types of implants (two expandable sinus tarsi implants and one calcaneal implant), reported comparable clinical and radiological outcomes with all three implant types [20]. Paolo et al. observed a greater improvement in foot mobility following the use of an endo-orthotic than a calcaneal implant [21]. A systematic review on arthoereisis showed a slightly lower rate of complications and better results with the use of calcaneal implants in comparison with other implant types [22].

We hypothesized that the use of a talar screw in patients with symptomatic planovalgus foot deformity would yield superior clinical and radiological outcomes.

Due to the lack of earlier studies on this topic, we aimed to conduct clinical and radiological assessments in patients with symptomatic pes planovalgus deformity treated with a talar screw.

## 2. Material and Methods

This study involved a prospective assessment of patients treated surgically for symptomatic flexible flatfoot deformity in the period 2021–2022. The study inclusion criteria were symptomatic flexible flatfoot, treatment via arthroereisis with the Spherus talar screw (Gruppo Bioimpianti S.R.L., Milan, Italy), age 7–14 years, informed assent, complete medical and radiological records, and a follow-up period of at least 12 months. The diagnosis of symptomatic flexible pes planovalgus was corroborated by the patient’s history, clinical examination, and radiological imaging. All evaluated patients had received conservative treatment, including rehabilitation and shoe inserts. All patients exhibited hindfoot eversion and medial longitudinal arch collapse and experienced foot pain and problems with gait. Study exclusion criteria were other lower limb pathologies (such as cerebral palsy, tarsal coalition, other foot deformities, or rheumatoid joint disease), history of foot surgery, neurological conditions, history of foot injuries, incomplete medical or radiological records, a lack of informed assent, or a follow-up period shorter than 12 months. This study was approved by the local ethics committee. All patients and their legal guardians were informed of the voluntary nature of their participation in this study. Thirty-five arthroereisis procedures with the use of the Spherus talar screw were conducted in our center in the years 2021 and 2022, Figure 1.

The application of the inclusion and exclusion criteria left 27 patients who were treated for pes planovalgus deformity as part of this study. The study group comprised 16 males and 11 females aged 7–14 years (mean age 10.5 years).

**Figure 2:**
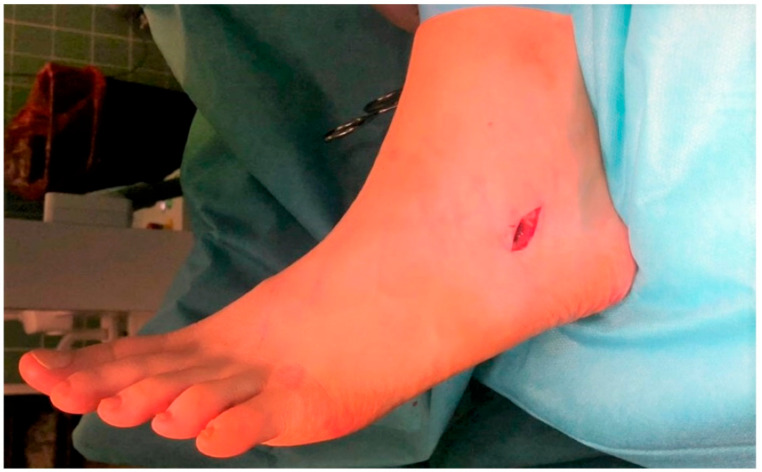
Intraoperative image of the surgical access site for Spherus screw insertion.

All study surgeries were performed by one of two experienced orthopedic surgeons and were conducted under general anesthesia and regional hemostasis. The patients were positioned prone, and an oblique, 1–2 cm-long incision was made along skin tension lines on the lateral aspect of the foot, at the level of the sinus tarsi, Figure 2.

Once the subcutaneous tissue was incised, the soft tissues of the sinus tarsi were dissected away with scissors to expose the inferior surface of the talus. With the foot maximally inverted and held at the right angle, as ensured via fluoroscopy, a pilot hole for the screw was created in the inferior surface of the talus using a straight awl. The awl was directed obliquely (medially and slightly proximally). Once the pilot hole was created, the appropriate screw was selected, with its length and diameter dependent on the patient’s age, the size of the patient’s talus, and the length of the patient’s foot. With the awl withdrawn and the patient’s foot held steadily at the right angle in maximum inversion, the selected screw was inserted into the prepared pilot hole. The screw was advanced, under fluoroscopy, until the desired degree of correction was achieved, with the spherical bulge of the screw protruding into the sinus tarsi and resting against its floor and walls. The surgical wound was then sutured and closed in layers. Concomitant lengthening of the Achilles tendon (Z-plasty) was performed in 36.36% of patients. The indication for Achilles tendon lengthening was a dorsiflexion of less than 5–10 degrees in the neutral position of the foot [1,2,5,14,15]. Full weight bearing was allowed on day one after surgery. In the case of concomitant Achilles tendon lengthening, the limb was immobilized in a short leg cast for 6 weeks. The patients were discouraged from strenuous exercise and sports during the first 6 weeks after surgery [7,11]. The rehabilitation protocol was the same for all patients and was initiated either at 6 weeks after surgery or on day one after surgery in patients with and without Achilles tendon lengthening, respectively. All patients were taught how to walk with crutches; the rehabilitation protocol also involved both passive and active exercises of the foot and the ankle joint.

All patients underwent a clinical examination and radiological imaging and completed a questionnaire.

The following parameters were assessed in this study: Meary’s angle, the Costa–Bartani angle, the calcaneal pitch angle, surgery duration, the length of hospital stay, patient satisfaction, patients’ retrospective willingness to consent to the treatment they received, postoperative complications, and the preoperative and postoperative use of analgesics.

Meary’s angle, measured on a lateral X-ray view of the foot, is the angle between the line drawn along the longitudinal axis of the talus (i.e., the mid-talar axis) and that along the longitudinal axis of the first metatarsal (i.e., the first metararsal axis) [4,7,15]. The Costa–Bartani angle, also measured on a lateral view of the foot, is the angle between the lines connecting three points: the lowermost point of the medial sesamoid, the lowermost point of the posterior calcaneal tuberosity, and the lowermost point of the talonavicular joint [4,7]. In order to mark the calcaneal pitch angle, a line was drawn from the inferior border of the distal articular surface of the calcaneus to the plantar surface of the calcaneus. The angle formed between this line and the transverse plane was the calcaneal pitch angle [4,7,15]. Postoperative complications were assessed based on the patients’ accounts, a review of records, clinical examination, and radiological imaging. The following complications were evaluated: persistent pain, limited range of motion, edema, infection, delayed wound healing, screw breakage or loosening, the lack of achieved correction, hypercorrection (i.e., a varus foot), and the need for reoperation or early implant removal. The duration of surgery was measured in minutes. The length of hospital stay was measured in days. The degree of patient satisfaction with the treatment was rated as “highly satisfied”, “satisfied”, or “dissatisfied”. We assessed how many patients would choose the same treatment method again and how many patients were taking analgesic agents (paracetamol, NSAIDs, and tramadol) prior to surgery and at the final follow-up visit.

### Statistical Analysis

Data were analyzed using the Statistica 13.1 software. The Shapiro–Wilk test was used to check for normality of distribution. The Student’s *t*-test and Mann–Whitney U test were used to compare quantitative variables. The level of statistical significance was set at *p* < 0.05.

## 3. Results

The mean follow-up period was 14.76 months (ranging from 12 to 26 months). Meary’s angle decreased from 18.63° before surgery to 9.39° at the final follow-up visit, Table 1. The difference was statistically significant (*p* = 0.004).

**Figure 3:**
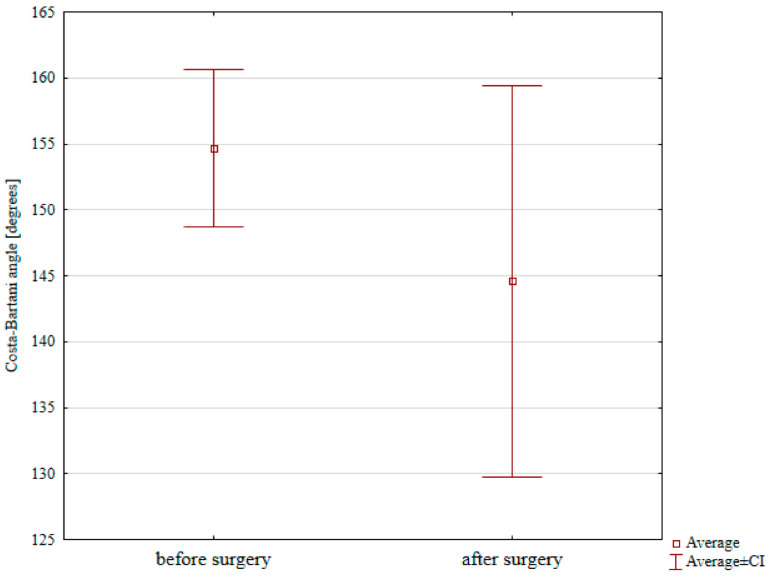
Preoperative and postoperative Costa–Bartani angle values.

Costa–Bartani angle decreased significantly from 154.66° before surgery to 144.58° after surgery, Table 1 and Figure 3 (*p* = 0.012).

The calcaneal pitch angle changed from 16.21° before to 19.74° after surgery; this difference was not significant, Table 1.

Complications were reported in three patients (11.11%). In each of these three cases, the nature of the complications was pain in the operated foot, which was treated with rehabilitation, shoe inserts, and ice packs. None of the patients experienced a limited range of motion, edema, infection, delayed wound healing, screw breakage or loosening, lack of deformity correction, hypercorrection (foot varus), the need of reoperation, the need of early screw removal, or any other complications.

The mean surgery duration was 32 min (SD 8.65). The mean hospital stay lasted 2.2 days (SD 0.41).

Fourteen patients (51.85%) were highly satisfied with the treatment, and 12 patients (44.44%) were satisfied with treatment, whereas one patient (3.7%) was dissatisfied with the treatment.

Twenty-five (92.59%) of the evaluated patients would choose the same type of treatment again.

Six patients (22.22%) needed to use analgesics prior to surgical treatment, whereas none of the patients needed to use them at final follow-up.

## 4. Discussion

This study assessed clinical and radiological parameters, both of which improved after surgical treatment of pes planovalgus with a talar screw, which supports our research hypothesis.

Due to its high prevalence, symptomatic pes planovalgus deformity in children is an important problem both for the patients themselves and the orthopedic surgeons [1,2,3,4,5,6,7,8,9,10,11,12,13,14,15,16,17,18,19,20,21,22]. There have been a number of reported surgical techniques for the treatment of pes planovalgus [1,2,3,4,5,6,7,8,9,10,11,12,13,14,15,16,17,18,19,20,21,22]. The lack of reports on the outcomes of talar screw placement through the sinus tarsi, including the placement of the Spherus talar screw, in patients with symptomatic pes planovalgus prompted us to study this topic. In assessing the outcomes of foot deformity treatment, it is important to consider multiple parameters, including both clinical and radiological aspects [1,2,3,4,8,9,10,14,15,16,17,18,21,22].

Flatfoot deformity is also diagnosed and treated in adults [23,24]. Like in children, the diagnosis is based on an X-ray under weight-bearing conditions [23,24]. Additionally, ultrasound may be used to assess any impairment of the tibialis anterior muscle and other soft tissues [23]. Computed tomography is used for detailed bone deformity assessment and preoperative and postoperative radiographic parameter analysis [23]. However, computed tomography scans are seldom used in children with symptomatic flexible flatfoot due to the high doses of ionizing radiation and the need to sedate very young children to perform the scan. Subtalar arthroereisis performed in adult patients has been reported to improve clinical and radiological outcomes [24]. However, unlike children, adults experience high rates of complication after treatment with this technique [24].

Jerosch assessed the outcomes of 21 arthroereisis procedures with a calcaneal screw [1]. The mean preoperative Meary’s angle of 162° significantly increased to 174° after surgery [1]. Kubo et al. analyzed the treatment of 95 patients with pes planovalgus [2]. The mean preoperative Meary’s angle of 18.9–19.5° decreased significantly to 13.3°–16.1° after the surgery [2]. Leonchuk et al. assessed 52 children after subtalar arthroereisis with the use of a fibular bone graft [3]. The mean preoperative and postoperative Meary’s angles in this population were 32.8° and 4.1°, respectively [3]. In another study, Arbab et al. evaluated 73 feet after arthroereisis procedures performed with a calcaneal screw [4]. In that study, the mean preoperative and postoperative Meary’s angles were 11.2° and 5.5°, respectively [4]. In another study, patients treated with expandable sinus tarsi implants achieved Meary’s angle reduction from the mean value of 22.4° before to 8.4° after surgery [5]. In a group of 84 feet assessed by Elmarhgany et al. Meary’s angle was 26.04° before and 3.3° after surgery [9]. Wang et al. reported a preoperative Meary’s angle of 24.4° and the postoperative angle of 5.3° [15]. In our study, we achieved a significant reduction in Meary’s angle from 18.63° preoperatively to 9.39° postoperatively, which is consistent with data from the literature [2,3,4,5,9,15].

Arbab et al. reported the mean Costa–Bartani angle of 133.1° preoperatively and 129.2° postoperatively [4]. Elmarhgany achieved a reduction in the mean Costa–Bartani angle from 151° to 127° [9]. A group of 68 patients evaluated by de Retana had the mean preoperative Costa–Bartani angle of 144.9° and the postoperative angle of 127.5° [14]. In our study, we achieved a significant reduction in the mean Costa–Bartani angle from 154.66° before to 144.58° after surgery, which is consistent with the data reported in the literature [4,9,14].

The mean calcaneal pitch angle in the group assessed by Kubo increased from 6.7°–12.7° before surgery to 8.8°–14.3° after surgery [2]. The group evaluated by Arbab et al. had the mean preoperative calcaneal pitch angle of 14.3° and the postoperative angle of 17.6° [4]. The patients assessed by de Bot et al. had the mean preoperative and postoperative calcaneal pitch angles of 11.7° and 14.5°, respectively [5]. Elmarhgany reported the calcaneal pitch angles of 6.97° and 23.9° before and after surgical treatment, respectively [9]. The preoperative and postoperative calcaneal pitch angles measured in a study by Wang et al. were 13.5° and 18.9°, respectively [15]. The increase in calcaneal pitch angle values from 16.21° preoperatively to 19.74° postoperatively observed in our study is consistent with the relevant data reported in the literature [2,4,5,9,15].

The reported complication rate following fibular bone graft was 6.9%, with one case (1.72%) of graft migration which required reoperation [3]. Arbab et al. observed complications in 1.3% of evaluated cases, with the nature of the complications including loss of correction and screw loosening [4]. Six out of 26 feet (23%) treated with Kalix II arthroereisis required revision surgery due to implant migration [5]. The rates of arthroereisis complications reported in the literature range from 4.8% to 18.6% [6]. Another literature review on arthroereisis with the use of bioabsorbable implants showed the rates of complications ranging from 2% to 65% [7]. Hong et al. reported complications in 20.5% of patients treated with an interference screw, with no cases of screw breakage or migration [8]. Elmarhgany reported complications in 3.57% of the operated feet [9]. The complications reported by Elbarbary et al. affected 4.35% of the evaluated group and included one case of infection that required implant removal after 4 months [10]. The complication rate in the group of 41 patients assessed by Franz et al. was 19.5% [12]. Martinelli reported complications in 10.2% of patients, with 6% of patients experiencing persistent pain that necessitated implant removal, 2% of patients experiencing residual deformity, and 2% of patients experiencing sports activity limitations [13]. De Retana reported complications in 33.8% of patients [14]. These included undercorrection (11.8%), overcorrection (7.4%), Achilles tendon contracture (5.9%), pain (5.9%), and peroneal tendon contracture (2.9%) [14]. Wang observed a 25.8% rate of complications in a group of 31 patients, with six patients reporting pain, one patient requiring implant removal, and one patient experiencing implant migration [15]. The rate of complications observed in our study was 11.11%. All complications were in the form of postoperative pain, which we consider to be a mild complication. In all cases, the pain resolved following rehabilitation and the use of shoe inserts and ice packs. We did not observe any serious complications, such as movement limitations, infection, delayed wound healing, screw breakage or loosening, lack of deformity correction, or hypercorrection (foot varus). Such serious complications have been reported by other authors [3,4,5,10,13,14,15]. Moreover, in our study, there were no cases requiring reoperation or implant removal. In light of these observations, our study results appear to be somewhat better than those reported in the literature [3,4,5,6,7,8,9,10,12,13,14,15,20]. With our study follow-up limited to short- and medium-term only; however, we are aware of the possibility of new complications emerging over the course of a longer follow-up. These might be more serious complications requiring additional treatment and, possibly, reoperation.

The mean duration of surgery reported by Elmarhgany was 20 min [9]. In another study, which involved subtalar arthroereisis, the mean duration of surgery was 46.1 min [15]. In our group of patients, the mean surgery time was 32 min, which is similar to the values reported in the literature [9,15] and indicates a relatively short surgery duration.

To date, there have been no studies evaluating the mean duration of hospital stay after an arthroereisis procedure. In our study, the mean hospital stay lasted 2.2 days, which indicates relatively short hospitalizations.

Ninety-five percent of patients who underwent arthroereisis with a calcaneal screw were either satisfied or highly satisfied with the results of treatment [4]. Overall satisfaction with treatment was also expressed by 81.25% of patients treated with expandable sinus tarsi implants [5]. Martinelli assessed 49 patients treated with expandable sinus tarsi implants and reported 89% of patients being satisfied with the treatment [13]. Out of 68 patients evaluated by de Retana, 91% were highly satisfied or satisfied with treatment [14]. In our study, 96.3% of patients reported being highly satisfied or satisfied with treatment, which is consistent with the proportions reported by other authors [4,5,13,14] and indicates good patient-reported treatment outcomes.

Ninety-six percent of patients assessed by Arbab et al. would choose the same treatment method once again if the need arose [4]. In our study, 92.59% of patients would choose the same treatment method again, which is consistent with the data reported by other authors [4].

There have been no available reports on the use of analgesics by patients undergoing arthroereisis. In our study group, 22.22% of patients used analgesics before the surgery; however, no patients needed analgesics after the surgery, which indicates good treatment outcomes and a reduced pain severity after treatment.

Arthroereisis is intended to limit subtalar joint pronation and to achieve a physiological position of the foot under weight-bearing conditions via inserting an implant into the sinus tarsi [1,2,7,10,13]. Implant size selection is very important for this procedure [7,14,15]. An implant that is too large may excessively limit subtalar joint mobility and cause pain, whereas an implant that is too small will not ensure a complete correction of the deformity and may become loose [7,14,15]. The exact mechanism in which arthroereisis improves foot function and biomechanics has not been fully explored [2]. Arthroereisis restricts calcaneal eversion, which limits calcaneal valgus [2,14]. The implants also improve proprioceptive perception [2]. The sinus tarsi contains many nerve endings, which makes implant insertion affect neurological proprioception as a result of foot anatomy alteration [2]. Overweight and ankle joint space valgus deformity may adversely affect arthroereisis treatment outcomes [2,3]. Our study showed that the introduction of the Spherus talar screw through the sinus tarsi helps achieve outcomes comparable with those achieved with calcaneal screws. Arthroereisis with the use of the Spherus talar screw permits active heel inversion but blocks excessive heel eversion, which limits such complications as implant loosening or migration [10].

The use of the Spherus talar screw seems to have several advantages over techniques involving calcaneal screws or sinus tarsi implants. First, there is a lower risk of screw loosening or migration. In our study, there were no cases of Spherus screw loosening or migration, whereas other authors reported such complications with calcaneal screws (loosening or migration rates of 1.72%) [3] and sinus tarsi implants (migration rates of 3.2% to 23%) [5,15]. The absence of reports of Spherus screw loosening or migration may be due to several factors, which are, first, the special conical shape of the screw, second, a relatively large diameter of the screw (in comparison with the diameters of calcaneal screws), and third, a large diameter of the thread facilitating screw introduction into cancellous bone; these structural characteristics of the Spherus screw ensure its larger contact area with the bone and its superior stability inside the bone in comparison with calcaneal screws. Moreover, screw introduction into the talus, instead of the calcaneus, may be associated with lesser forces acting on the screw during full weight bearing. Another advantage of using the Spherus screw is the fact that it does not have to be removed over the mean follow-up period of 14.76 months. Conversely, arthroereisis with the use of calcaneal screws or sinus tarsi implants required implant removal due to pain, inflammation, or implant migration in 1.2%–40% of patients after 4 months–8 years [3,5,6,7,9,10,13,14,15,20]. All patients after subtalar arthroereisis with fibular bone graft required another surgery to remove the Kirschner wire from the foot after a mean period of 7 months following the first surgery [3].

The time to implant removal and the need for its removal after subtalar arthrodesis has not been unequivocally established [6,7,14]. Some authors suggest leaving the implant in for 2–3 years, which is the time required for adequate soft tissue and bone adaptation [6,7,14]. Following surgery with the use of the Spherus talar screw, patients were allowed to walk with full weight bearing on day one after surgery. Conversely, some of the other surgical techniques require partial weight bearing for 2–8 weeks and the use of a cast for 2–8 weeks [3,5,13,14,15].

Spherus screw arthroereisis is a very simple technique, and it allows early weight bearing, uses a low-cost implant, and requires neither a large surgical access nor precise tissue dissection in comparison with other techniques [3,8]; the mean surgery duration is 32 min. Some authors have reported talus avascular necrosis following arthroereisis [3]; however, this complication was not observed in our study group. Subtalar arthroereisis with the Spherus screw does not disrupt bone growth, which is beneficial in case of the potential need for osteotomy in the future [15].

Some authors described arthroereisis performed simultaneously with other surgical procedures of the foot and ankle joint [1,2,3,8,10,12,14,15]. The most common concomitant procedures (performed in 17%–100% of patients) were Achilles tendon lengthening and gastrocnemius lengthening [1,2,3,5,10,12,14,15]. Achilles tendon and gastrocnemius contractures are common in patients with flexible flatfoot. We believe that all cases of Achilles tendon contracture require a tendon-lengthening procedure. In our study, 36.36% of patients required Achilles tendon lengthening due to contracture. A study by Wang suggests that the additional surgical procedure does not affect either the radiological or clinical outcomes of subtalar arthroereisis [15].

The goal of pes planovalgus treatment is to improve clinical and radiological parameters [2,5,6,7,8,9,10,19]. In our study, 96.3% of patients were highly satisfied or satisfied with treatment, and 92.59% of patients would choose the same treatment again. Moreover, the assessed radiological parameters improved after talar screw surgery, which suggests good treatment outcomes.

Limitations of our study include a relatively small sample size, which is a result of our desire to conduct prospectively both the clinical and radiological assessments. Nonetheless, other authors evaluated patient groups of similar size [1,5,8,10,15,16,17,19,21]. Another limitation of our study was the mean follow-up duration; however, as reported in the literature, most patients had normal foot function at four weeks after arthroereisis [11]. Some of the other, similar studies had a follow-up period similar to that in our study [11,15,16,21]. Due to its prospective nature, our study had no control group. We are planning to conduct another study and this time with a control group.

The strengths of our study are its prospective design (while some of the earlier studies were retrospective) [1,2,5,8,13,15,16,19,20], the fact that all surgeries were performed by one of only two experienced orthopedic surgeons, and the fact that both radiological and clinical parameters were assessed. We are planning a future study in a larger group of patients treated with the use of a talar screw, with a longer follow-up. Another one of our studies, whose results are soon to be published, involved gait assessment in a group of patients of similar size.

## 5. Conclusions

The use of the Spherus talar screw is a simple, minimally invasive, and effective method of treating symptomatic flexible flatfoot.

Spherus screw arthroereisis helps improve radiological parameters of patients with flexible flatfoot.

We observed good clinical outcomes after treatment with a talar screw, with a majority of patients reporting being satisfied or highly satisfied with treatment.

The short- and medium-term treatment outcomes of pes planovalgus treatment with the use of the Spherus screw are good.

## Figures and Tables

**Figure 1:**
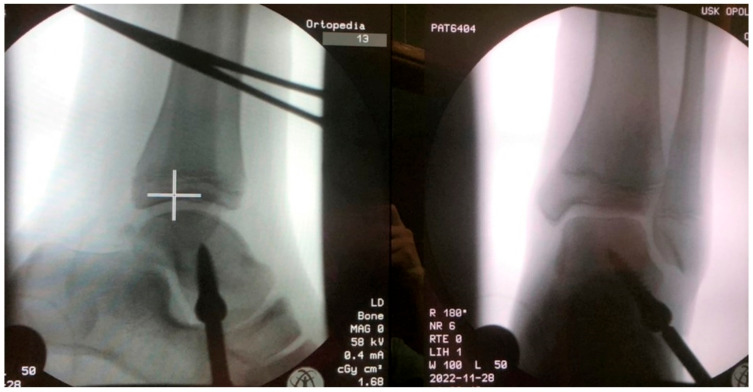
Radiologic images after pes planovalgus correction with the Spherus screw.

**Table 1:** Radiological parameters before and after surgery.

Analyzed Variable	Before Surgery	After Surgery	*p* Value
	Mean ± Standard Deviation		
Meary’s angle (degrees)	18.63 ± 7.71	9.39 ± 6.85	0.004 *
Costa–Bartani angle (degrees)	154.66 ± 5.98	144.58 ± 14.83	0.012 **
Calcaneal pitch (degrees)	16.21 ± 4.41	19.74 ± 7.33	0.275 **

* Student’s *t*-test; ** Mann–Whitney U test.

## Data Availability

The data presented in this study are available on request from the corresponding author. The data are not publicly available due to privacy.
